# Clinical Outcomes of Catheter-Directed Thrombolysis versus Mechanical Aspiration in Patients with Acute Pulmonary Embolism

**DOI:** 10.5761/atcs.oa.25-00012

**Published:** 2025-04-11

**Authors:** Zhe Zhang, Shanshan Jin, Bin Liu, Hai Feng, Wenrui Li

**Affiliations:** Department of Vascular Surgery, Beijing Friendship Hospital, Capital Medical University, Beijing, China

**Keywords:** acute pulmonary embolism, catheter thrombolysis, suction thrombectomy, aspiration thrombectomy

## Abstract

**Purpose:** The objective of this study was to evaluate the safety and efficacy of catheter-directed thrombolysis (CDT) and mechanical aspiration (MA) for acute pulmonary embolism (PE).

**Methods:** From February 2022 to October 2024, the clinical data of patients with high- and intermediate-risk PE who received endovascular therapy were retrospectively reviewed. Patients were categorized based on the treatment strategy.

**Results:** Fifty-eight consecutive patients were identified. CDT was initiated in 29 patients, while the remaining 29 received MA treatment. The time of thrombolysis and the dosage of urokinase were both lower in the MA group (P <0.05). No differences were found in cardiac biomarkers after 48 hours, perioperative bleeding events, heart/valve injury, and mortality. The total cost of the MA group was much higher compared to CDT alone. The MA group showed better improvement in right ventricular (RV) function with a higher reduction in the right ventricular-to-left ventricular ratio (0.55 ± 0.46 vs. 0.13 ± 0.53, P = 0.017). No differences were found in the reduction of the CT obstruction index.

**Conclusion:** CDT and MA seem to have similar outcomes for patients with acute high- and intermediate-risk PE. MA is more effective in improving RV function with less thrombolysis time and fewer thrombolytics.

## Introduction

Pulmonary embolism (PE) is a common and potentially fatal disease, with incidence rates ranging from 39 to 115 cases per 100000 people annually.^1)^ It remains a significant cause of cardiopulmonary disability and mortality that can vary substantially.^2,3)^ The outcomes in acute PE depend on the severity of patient characteristics, which are categorized as high-, intermediate-, and low-risk.^4)^

Generally, guidelines recommend these percutaneous treatments as alternatives to systemic thrombolysis (ST) in patients who have deteriorated hemodynamically or are at risk for cardiopulmonary instability despite initial anticoagulation.^5)^ On one hand, percutaneous treatments of PE are considered to decrease clot burden, prevent mortality, and counter the bleeding risks of ST.^4,6,7)^ On the other hand, catheter-directed treatments are targeted to decrease recurrence and any potential late-onset PE complications such as chronic thromboembolic pulmonary hypertension.^3)^

Catheter-directed thrombolysis (CDT), ultrasound-assisted thrombolysis, and percutaneous mechanical aspiration (MA) have been used in different clinical trials and have shown an advantage in early cardiac function recovery compared with anticoagulation.^6,8–10)^ Among them, MA devices were used to achieve percutaneous thrombectomy through sustained suction with or without the use of thrombolytics. The FlowTriever aspiration device (Inari Medical, Irvine, CA, USA) showed a reduction in right ventricular-to-left ventricular (RV:LV) ratio in intermediate-risk PE in the single-arm FLARE (FlowTriever for acute massive PE*)* trial, and the EXTRACT-PE (Evaluating the Safety and Efficacy of the Indigo aspiration system in Acute PE) trial used the smaller-bore Indigo aspiration catheter (Penumbra, Alameda, CA, USA) to achieve a significant reduction in the RV:LV ratio as well.^6,11)^ However, the effectiveness and safety of these MA devices have not yet been studied systematically. The goals of this study were to compare clinical outcomes of these different interventional therapies.

## Materials and Methods

This study was an institutional review board-approved study evaluating different endovascular treatments for acute PE. A single-institution procedural database was queried for all consecutive patients who underwent CDT or MA for acute high- and intermediate-risk PE from February 2022 through October 2024. This study was conducted in accordance with the principles of the Declaration of Helsinki.

PE types were classified in accordance with established published guidelines. All patients were diagnosed with acute PE based on clinical presentation, computed tomography angiography (CTA), and D-dimer levels. Patients with a history of chronic PE or chronic pulmonary hypertension were excluded. High-risk PE is characterized by sustained hypotension for a minimum duration of 15 minutes or the requirement for vasopressor support. Intermediate-risk PE is characterized by the presence of RV dysfunction as evidenced on echocardiography or CT scans, and/or positive cardiac biomarkers in patients who were hemodynamically stable. We excluded patients with acute PE treated with anticoagulants alone or ST, patients with incomplete medical records or insufficient imaging data, and patients exhibiting allergies to anticoagulants or thrombolytic agents.

The patients were divided into 2 groups: Group A was treated with CDT alone; Group B was treated with MA combined with CDT or not.

### Treatment strategy

**Fig. 1:**
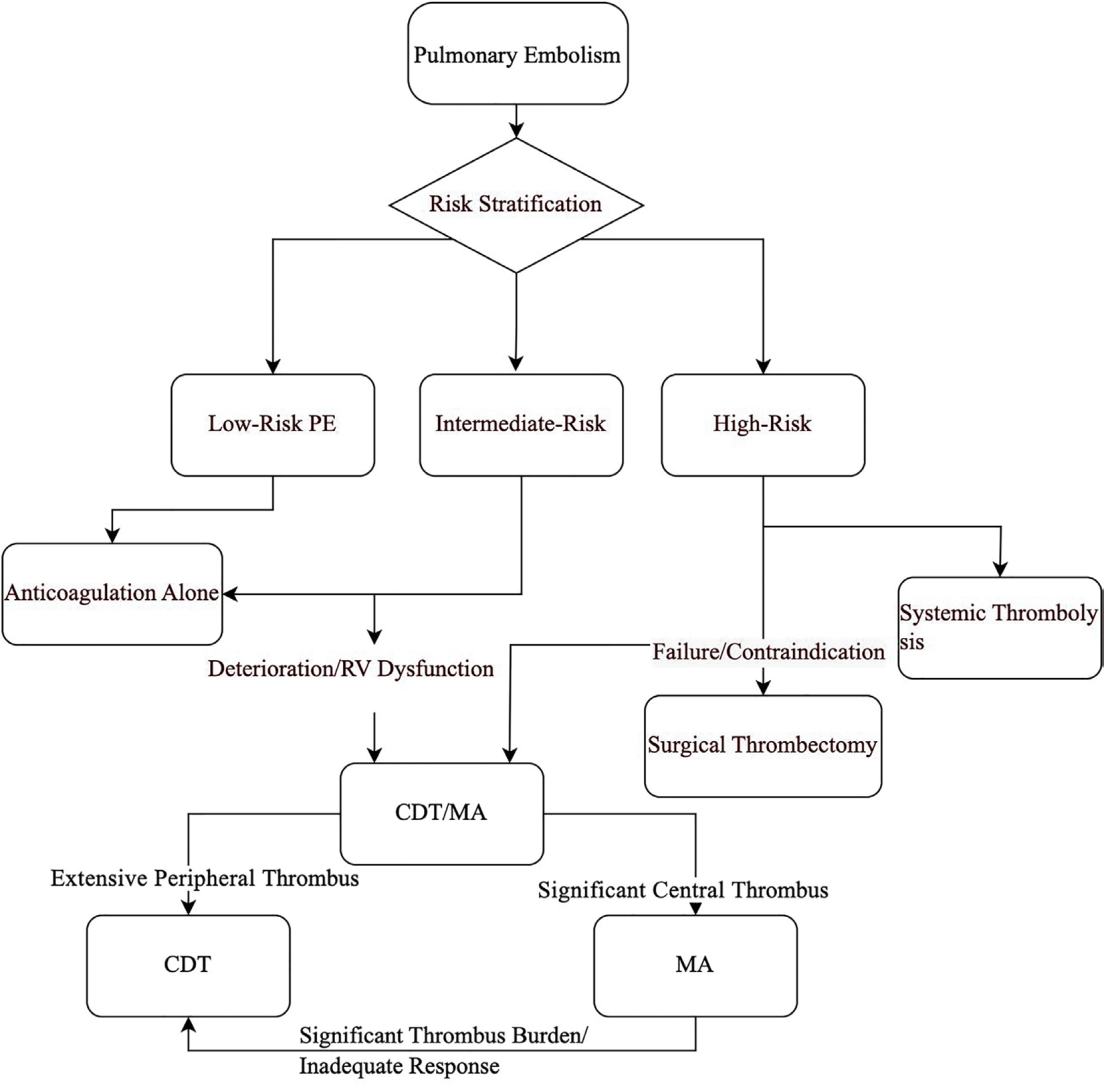
A simplified management pathway for acutePE. CDT: catheter-directed thrombolysis; MA: mechanical aspiration; PE: pulmonary embolism; RV: right ventricular

Immediate anticoagulation was initiated with low-molecular-weight heparin at a dosage of 90 IU/kg of body weight, administered twice daily. Before the procedure, we inserted a retrievable inferior vena cava (IVC) filter (Denali IVC filter [Bard Peripheral Vascular, Tempe, AZ, USA] or Option [Argon Medical, Frisco, TX, USA]) was usually implanted through the contralateral femoral vein or right internal jugular vein. The choice of using MA depended on the extent of PE, the comorbidities, and the hemodynamic conditions. In detail, patients were more likely to be selected for MA if they had significant thrombus burden, hemodynamic instability, or high bleeding risk (**Fig. 1**). No patients underwent surgical thrombectomy during the study period.

Pulmonary angiography was performed before intervention in all cases with a pigtail catheter (Cordis Corp, Fremont, CA, USA). For patients receiving CDT alone, the procedure was performed using 1 or 2 multiple-sided hole infusion catheters (Multi-Sideport; Cook, Bloomington, IN, USA) via the femoral vein with urokinase at a dosage of 200000–800000 IU/day. Thrombolysis was monitored by measuring fibrinogen levels (with a fibrinogen concentration greater than 1 g/L). Pulmonary angiography was performed to assess the efficacy of thrombolysis, and the thrombolysis treatment was discontinued 24 to 96 hours after the initial procedure.

In patients treated with MA, it was performed with the Indigo Catheter (8 Fr) (Penumbra), which was connected to a specially matched suction pump that exerted negative pressure (20–40 cm H_2_O). An exclusive wire separator (SEP, Penumbra) was used at the same time to clear the catheter. MA was terminated when the operator confirmed a substantial reduction in thrombus burden (estimated reduction of approximately 50%–70%). For patients with significant thrombus burden in small branches or inadequate response to MA (less than 50%), additional CDT was administered.

After discharge, patients received anticoagulant therapy with factor Xa inhibitors (rivaroxaban or edoxaban) or vitamin K antagonists (therapeutic target international normalized ratio of 2:3), with the anticoagulant timing and dose consistent with published guidelines.^4)^

### Study endpoints and definitions

For the diagnostic evaluation of RV function and the extent of thrombosis, we measured several rule-based criteria based on vessel measurements from contrast-enhanced chest CTA according to previous research. This included the ratio of the main pulmonary artery diameter to the aortic diameter (rPA), measured at the level of the bifurcation of the main pulmonary artery,^12)^ and the RV/LV diameter ratio. The LV and RV end-diastolic diameters were provided in the original database and measured using a cardiac 4-chamber view.^13)^ Additionally, the clot burden was measured with the Qanaldi CT obstruction index according to previous research standards.^14)^

The primary efficacy endpoints were the changes in the Qanadli CT obstruction index, RV/LV diameter ratio, and rPA after treatment. Secondary endpoints included changes in cardiac biomarkers, survival to hospital discharge, and the dosage of urokinase used. Safety outcomes were procedure-related complications and bleeding events within 72 hours after the procedure. The severity of bleeding events was defined in accordance with the Global Utilization of Streptokinase and t-PA for Occluded Coronary Arteries (GUSTO) classification system. Within this framework, both GUSTO moderate and GUSTO severe bleeding events were classified as major bleeding.^15)^

Statistical analyses were performed using the Statistical Package for Social Sciences (SPSS version 24.0; SPSS, Chicago, IL, USA). Continuous variables were reported as means ± standard deviation or median and interquartile range (25th–75th percentiles). Comparison between means was conducted using Student’s t-test for unpaired data, whereas comparison between frequencies was performed using the chi-square test and Fisher’s exact test, respectively. Risk assessment was carried out by calculating odds ratios with 95% confidence intervals. The null hypothesis was rejected at a 2-tailed P-value <0.05.

## Results

Over the 2-year study prioed, we identified 58 acute PE patients who underwent endovascular surgery with CDT or MA. MA was performed in 29 (50%) patients, and the remaining patients received CDT alone. The mean patient age was 67 ± 13 years, and 52% were men. The median time from symptom onset to treatment was 3 days. The patients’ demographic data, clinical presentations, and characteristics are shown in **Table 1**. The mean CT obstruction index at baseline was 49.8 ± 16.7. In general, more patients had a history of venous thromboembolism (VTE) in the CDT group, with 8 patients having a history of VTE; all of them had stopped oral anticoagulants before PE onset. The MA group had more serious PE with a higher baseline CT obstruction index and RV/LV ratio, indicating worse RV dysfunction. However, there were no significant differences in PE types between the CDT group and the MA group. In the MA group, 17 (58.6%) patients received CDT after aspiration. The treatment details and complications are shown in **Table 2**. The procedure duration of CDT was slightly shorter than in the MA group but without a significant difference (80 vs. 95 minutes; P >0.05). The time for thrombolysis and the dosage of urokinase were both lower in the MA group. No differences were found in cardiac biomarkers after 48 hours, perioperative bleeding events, heart/valve injury, and mortality between the 2 groups. Major bleeding occurred in 5 patients: in the MA group, 2 experienced gastrointestinal bleeding within 24 hours postoperatively and 1 experienced heart injury-related hemorrhage. In the CDT group, there was 1 case of postoperative retroperitoneal hematoma and 1 case of gastrointestinal bleeding. Two cases (6.9%) of heart/valve injury complications occurred in the MA group; both patients showed hypotension after the operation, and echocardiography confirmed a large amount of pericardial effusion. Pericardial puncture and drainage were performed immediately; 1 patient recovered well, while the other patient died. One patient in the MA group died of respiratory infection and failure 2 days after the operation. The total cost of the MA group was much higher compared to CDT alone (83173 ± 19289 Yuan vs. 55074 ± 12935 Yuan; P <0.05).

**Table 1:** Demographic and clinical information of patients stratified by treatment

Variables	All patients (n = 58)	CDT alone (n = 29)	MA (n = 29)	P ^*a*^
Age, mean ± SD, years	67.0 ± 12.7	66.8 ± 12.9	67.0 ± 14.9	0.955
Male, %	30 (51.7)	17 (58.6)	13 (44.8)	0.293
Active smoking, %	8 (13.8)	5 (17.2)	3 (10.3)	0.703
Comorbidities, %				
Hypertension, %	30 (51.7)	16 (55.2)	14 (48.3)	0.599
Diabetes mellitus, %	9 (15.5)	5 (17.2)	4 (13.8)	1.000
Coronary artery disease, %	7 (12)	2 (6.9)	5 (17.2)	0.420
Chronic renal insufficiency, %	2 (3.4)	1 (3.4)	1 (3.4)	1.000
Any cancer, %	8 (13.8)	4 (13.8)	4 (13.8)	1.000
Previous VTE, %	8 (13.8)	8 (27.6)	0 (0)	0.009
Duration of symptoms onset to treatment, median (IQR), d	3 (1–3.2)	3 (1.5–3.5)	2 (1–4)	0.184
WBC count, median (IQR), ×10^3^/dL	7.9 (6.3–9.9)	7.0 (6.1–9.1)	9.0 (7.1–10.2)	0.028
RBC count, mean ± SD, ×10^3^/dL	4.5 ± 0.6	4.5 ± 0.7	4.4 ± 0.6	0.552
Plt count, mean ± SD, ×10^3^/dL	200 ± 76	205 ± 58	193 ± 88	0.535
Creatinine, median (IQR), μmol/L	71.5 (61.3–92.6)	70.5 (58.5–91.3)	72.6 (63.5–93.8)	0.392
D-dimer (IQR), mg/L	7.6 (3.9–12.4)	5.8 (2.9–10.7)	9.2 (5.5–14.6)	0.086
Baseline CT obstruction index, mean ± SD	49.8 ± 16.7	43.5 ± 18.2	55.2 ± 11.5	0.005
Baseline rPA, mean ± SD	0.91 ± 0.12	0.91 ± 0.14	0.91 ± 0.11	0.793
Baseline RV/LV, mean ± SD	1.32 ± 0.36	1.18 ± 0.31	1.44 ± 0.35	0.003
Hypoxemia^*^, %	12 (20.7)	6 (20.7)	6 (20.7)	1.000
Elevated NT-proBNP^*^, %	24 (41.4)	10 (34.5)	14 (48.3)	0.286
Elevated troponin I^*^, %	39 (67.2)	17 (58.6)	22 (75.9)	0.162
Elevated troponin T^*^, %	30 (51.7)	11 (37.9)	19 (65.5)	0.036
High-risk PE	4 (6.9)	1 (3.4)	3 (10.3)	0.604

P-value <0.05 is considered statistically significant.

^*a*^ Denotes comparisons between different treatments.

^*^Defined by laboratory values of >1800 ng/L (NT-proBNP), 0.03 ng/mL (troponin I), 0.017 ng/mL (troponin T), and PaO_2_ <60 mmHg or PaO_2_/FiO_2_ <300 mmHg.

CDT: catheter-directed thrombolysis; MA: mechanical aspiration; SD: standard deviation; VTE: venous thromboembolism; IQR: interquartile range; WBC: white blood cell; RBC: red blood cell; Plt: platelet; CT: computed tomography; rPA: the ratio of main pulmonary artery diameter to the diameter of the aorta; RV: right ventricular; LV: left ventricular; NT-proBNP: N-terminal pro B-type natriuretic peptide; PE: pulmonary embolism

**Table 2:** Comparison of treatment details and complications

Variables	CDT alone (n = 29)	MA (n = 29)	P
Procedure duration, median (IQR), min	80 (60–105)	95 (90–120)	0.107
Time of thrombolysis, median (IQR), h	72 (48–96)	24 (0–60)	<0.001
Dosage of urokinase, median (IQR), ×10^4^ IU	150 (100–185)	30 (0–100)	<0.001
Hospital stay median (IQR), day	8 (7–11.5)	8 (7–12.5)	1.000
Elevated NT-proBNP^*^ after 48 h, %	10 (34.5)	12 (41.4)	0.588
Elevated troponin I^*^ after 48 h, %	21 (72.4)	25 (86.2)	0.195
Elevated troponin T^*^ after 48 h, %	13 (44.8)	18 (62.1)	0.188
Major bleeding events, n (%)	2 (6.9)	3 (10.3)	1.000
GUSTO moderate, n (%)	2 (6.9)	2 (6.9)	1.000
GUSTO severe, n (%)	0 (0.0)	1 (3.4)	1.000
Heart/valve injury, n (%)	0 (0.0)	2 (6.9)	0.472
Death, %	0 (0.0)	2 (6.9)	0.472
Total cost, mean ± SD, yuan	55074 ± 12935	83173 ± 19289	<0.001

P-value <0.05 is considered statistically significant.

^*^ Defined by laboratory values of >1800 ng/L (NT-proBNP), 0.03 ng/mL (troponin I), 0.017 ng/mL (troponin T), and PaO_2_ <60 mmHg or PaO_2_/FiO_2 _ <300 mmHg.

CDT: catheter-directed thrombolysis; MA: mechanical aspiration; IQR: interquartile range; NT-proBNP: N-terminal pro B-type natriuretic peptide; GUSTO: Global Utilization of Streptokinase and t-PA for Occluded Coronary Arteries; SD: standard deviation

**Fig. 2:**
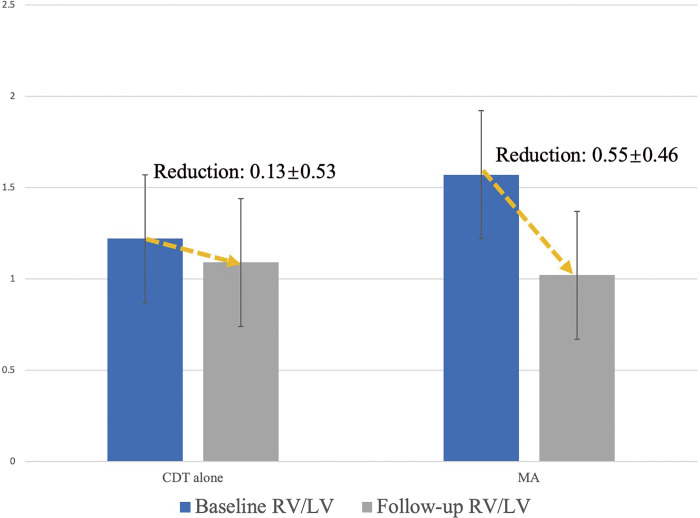
Mean reduction in RV/LV ratio by treatment modality. CDT: catheter-directed thrombolysis; LV: left ventricular; MA: mechanical aspiration; RV: right ventricular

A total of 35 patients completed follow-up with CTA, with a mean follow-up of 2.7 ± 2.2 months. The mean time to follow-up CT was 2.8 ± 2.1 months in the CDT group and 2.5 ± 2.1.5 months in the MA group. The results of follow-up in different treatment modalities are shown in **Table 3**. In general, the MA group had more serious PE compared to the CDT group, with a higher RV/LV ratio (1.57 ± 0.34 vs. 1.22 ± 0.35, P = 0.005), and the baseline CT obstruction index rates appeared higher for MA, but the difference did not reach significance (57.0 ± 11.7 vs. 47.5 ± 15.9, P = 0.055). The MA group showed better improvement in RV function with a higher reduction in the RV/LV ratio (0.55 ± 0.46 vs. 0.13 ± 0.53, P = 0.017) (**Fig. 2**). No differences were found in the reduction of the CT obstruction index and rPA between groups.

**Table 3:** Radiological outcomes by treatment modality

Variables	Overall (n = 35)	CDT alone (n = 19)	MA (n = 16)	P ^*a*^
Baseline CT obstruction index	51.9 ± 14.7	47.5 ± 15.9	57.0 ± 11.7	0.055
Follow-up CT obstruction index	16.1 ± 17.7	16.0 ± 19.1	16.2 ± 16.5	0.974
Reduction in CT obstruction index	35.7 ± 19.4	31.4 ± 20.9	40.8 ± 16.8	0.160
Baseline rPA	0.93 ± 0.12	0.95 ± 0.13	0.91 ± 0.10	0.357
Follow-up rPA	0.91 ± 0.12	0.82 ± 0.13	0.79 ± 0.10	0.477
Reduction in rPA	0.12 ± 0.14	0.12 ± 0.15	0.11 ± 0.11	0.851
Baseline RV/LV	1.38 ± 0.38	1.22 ± 0.35	1.57 ± 0.34	0.005
Follow-up RV/LV	1.06 ± 0.37	1.09 ± 0.41	1.02 ± 0.32	0.604
Reduction in RV/LV	0.32 ± 0.54	0.13 ± 0.53	0.55 ± 0.46	0.017

P-value <0.05 is considered statistically significant.

^*a*^ Denotes comparisons between different treatments.

CDT: catheter-directed thrombolysis; MA: mechanical aspiration; CT: computed tomography; rPA: the ratio of main pulmonary artery diameter to the diameter of the aorta; RV: right ventricular; LV: left ventricular

## Discussion

PE remains a substantial global threat to health despite considerable advancements made in the diagnosis and management in recent decades.^16)^ In particular, acute large-scale PE leads to pulmonary vascular resistance and consequent acute pulmonary artery hypertension and RV dysfunction.^17)^ Although several studies have demonstrated that advanced therapies improve RV function more quickly than anticoagulation alone, the optimal use of these catheter-based therapies remains uncertain.^3,6,18,19)^ In this single-center retrospective study, we included 58 patients with acute PE. These patients were treated with CDT or MA based on anticoagulation therapy. The perioperative complications and treatment outcomes were compared.

In general, both CDT and MA followed by CDT were effective and safe in treating patients with high- and intermediate-risk PE. The overall survival rate was 96.6%, which is quite acceptable given the relatively high CT obstruction index at baseline (49.8 ± 16.7). Similar results were found in the prospective ULTIMA (Ultrasound Accelerated Thrombolysis of Pulmonary Embolism) trial, PERFECT (Pulmonary Embolism Response to Fragmentation, Embolectomy, and Catheter Thrombolysis) registry, and EXTRACT-PE trial, which have shown to result in improved clinical outcomes and early RV function recovery in patients with acute PE.^6,8,20)^ Although no advantages were found in mortality in the MA group, this more proactive treatment showed better improvement in the RV/LV ratio compared to CDT alone. In previous studies, the effectiveness of this technique was evaluated, and the RV/LV ratio was improved remarkably, as well as the pulmonary artery systolic pressure.^19,21,22)^ These results showed that continuous MA was a promising treatment to decrease thrombus burden and reduce right heart strain. However, the improvement in the extent of thrombus (CT obstruction index) was not significant compared to the RV/LV ratio. We believe that this difference arises from the inadequacy of the Qanadli CT obstruction index in evaluating saddle embolus, which is precisely the advantage of MA. Therefore, for patients with a significant thrombus burden, CDT should be considered to reduce residual thrombus in small branches after initial MA to remove more centrally located thrombus.

Another potential advantage of MA, besides effective thrombus resolution, is reducing thrombolysis, or even offering it as a complementary option for patients with contraindications to thrombolytics in a minimally invasive approach. In our study, the MA group showed advantages in both thrombolysis time and dosage of urokinase. However, this superiority has not been reflected in the bleeding rate. Overall, the incidence of major bleeding in MA varied greatly from 1.8% to 16.7%.^3,19,22)^ We believe that the excellence of MA devices in reducing bleeding events needs to be verified in larger studies focused on patients who have contraindications to thrombolysis, as several studies have shown that this novel suction thrombectomy device is a promising technique when used alone.^6,21)^ Besides, open surgical treatment may be considered as a last resort for patients with persistent hypotension, cardiogenic shock, or severe right heart dysfunction despite CDT or MA.

Several potential drawbacks of these advanced therapies remain challenging. First, the larger and more complex devices lead to an increased risk of vessel injury, including pulmonary artery injury, cardiac injury, and cardiac tamponade. Although the risk for cardiac injury is minimal according to past studies, the consequences can be catastrophic once they occur.^6,22)^ For patients who develop hypotension postoperatively, cardiac tamponade should be vigilantly monitored, and immediate pericardial puncture and drainage are necessary upon diagnosis. Second, aspiration inevitably leads to blood loss, which can be significant when the catheter is not embedded in clots. In most cases, the blood loss is acceptable, and no patient required transfusion due to intraoperative blood loss in our study. However, this rapid blood loss may force the surgeon to prematurely terminate the thrombus aspiration, thereby affecting treatment efficacy. Therefore, future improvements are needed to return blood to the patient during aspiration. Lastly, the cost of newer devices should be considered in the era of cost-effective medicine. The total cost of the MA group was higher due to the use of additional devices, but this did not result in a reduction in hospitalization time or other treatments. However, our data are insufficient for a thorough analysis of long-term costs, as the improved recovery of RV function may bring more benefits and reduce medication or hospitalization costs in the future.

The major limitations of this study include its retrospective nature and small sample size. Second, the outcomes were based on short-term follow-up, and some patients had no follow-up CTA results, which may affect the actual outcomes. Third, the operators varied among different patients, which may lead to bias and influence the results. Larger, controlled studies are needed to validate these findings.

## Conclusion

In conclusion, CDT and MA are both safe treatments for patients with acute high- and intermediate-risk PE, with similar complication rates and mortality. MA is more effective in improving RV function with less thrombolysis time and fewer thrombolytics. The risk of vessel injury during surgery should be a concern. Future evaluations of these techniques and outcomes should continue to be pursued.

## Declarations

### Consent for publication

The patients/participants provided their written informed consent to participate in this study.

### Ethics approval

The studies involving human participants were reviewed and approved by the Institutional Ethical Review Board of Beijing Friendship Hospital (Review number: BFHHZS20240073).

### Funding

This work was supported by Beijing Friendship Hospital, Capital Medical University (No. YYZZ202205).

### Author contributions

Equal contribution: ZZ and SJ. Conception and study design: ZZ, SJ, and WL. Writing the article: ZZ and SJ. Critical revision: BL, HF, and WL. Data collection, analysis, and interpretation: WL. All authors contributed to the article and have read the manuscript and approved the final version of the manuscript.

### Disclosure statement

The authors declare that they have no competing interests.
